# The Howard Association

**Published:** 1896-11-28

**Authors:** 


### THE HOWARD ASSOCIATION.

There can be no question that the late Lord Brougham performed an excellent work when, in the year 1866, he founded an association, under the name of the great prison reformer, Howard, for the promotion of the best methods of penal treatment and crime. Very valuable results have followed the labours of this association, which does not confine itself to strictly penological questions but deals also with pauperism, intemperance, and the care and education of children whose maintenance falls upon the State. The list of subjects dealt with in the report under our notice is sufficient to show how widely extended is the range of the association's philanthropic operations: "Pauperism and Boarding Out, Children's Department, A Better Way, Probation, Reformatories, Education, Prison Officers, Sentences and Time for Reformation, Fines, Recent Complaints, The Authorities, Court Houses, The Death Penalty, France, Italy, United States, Penological Principles." We entirely agree with the association that the boarding-out system as practised with special success in Sheffield is infinitely preferable to barrack schools and reformatories. With regard to these last we have good reason to know that evils which are quite unavoidable in those establishments render them, in fact, more dangerous as regards contamination for young offenders than a committal to prison with its strictly enforced rules could possibly be. In the freer intercourse of the reformatories one vicious lad can exercise a most corrupting influence over his companions, and secret vice may be carried on in ways impervious to the scrutiny of the authorities. The boarding-out system does not, however, deal with criminal children, but with the hapless progeny of paupers or of the lowest class of neglectful and unnatural parents. We fully share the satisfaction of the association in the appointment of a departmental committee of the Local Government Board to inquire into this most important subject, which has already drawn forth very valuable evidence respecting it. We also highly approve of their suggestion that a special children's department, to take the oversight of the elder boys' training, the boarding out of girls and younger boys, and the definite care of idiotic and physically infirm children, should be established in the Local Government Board. Apart from much more important advantages, the boarding-out system is greatly less expensive than the barrack schools or cottage homes. We are also strongly of opinion that the care of the State in these matters should be extended to illegitimate children as well as to orphans and deserted children. Doubtless these unhappy infants, who can claim no legal rights of parental care, are the offspring of vice, and there is danger that by benefiting them in every way we are giving encouragement to immorality; but they have their rights as living and sentient beings, and considering how widespread is the range of immorality over the whole country, it may be questioned whether greater mercy shown to the innocent children whom it breeds would cause any appreciable difference on the extent of the evil. At present illegitimate children are kept for years in the workhouse, when they might reasonably share the advantages of the boarding-out system. While strongly advocating this plan the Howard Association recommends, with much earnestness, what the secretary terms "a better way," viz., the "enforcement of parental responsibility far more than has hitherto been done." This is carried out in America by what is named the "Probation Plan," and in the report it is thus described: "Probation officers are appointed to look after both neglected children and negligent parents . . . and by a little friendly counsel and help for the willing, and by small fines or a few days' imprisonment for the unwilling, many a lazy and drunken parent . . might be prevented from fastening his offspring on the backs of the rate and tax payers." Doubtless if this system could be made to work practically and effectually for the desired results, it would be of inestimable value to the community, but we must own to grave doubts whether it is not too Utopian a scheme to be at all generally successful in this country whatever it may be in Massachusetts, from whence the idea is taken. We judge thus from personal experience of the great numbers of men and women—chiefly the latter—who are sent to prison for shameless neglect of their children, often involving their deaths, and carried to an extent which we believe would be quite beyond any possible supervision by probation officers. The Society for the Prevention of Cruelty to Children has done much to bring such cases to judgment, but the efforts of the agents employed have shown only too plainly how almost hopelessly extensive the evil really is. It is also probably a matter of personal experience to all workers among the poor, how many parents in that class do their utmost to get rid of specially troublesome or burdensome children by any means in their power. They do their best to get them sent to homes or reformatories, and will readily sign papers giving up all their rights over them for the whole course of their lives. We fear that such persons, and they are extraordinarily numerous, would not be influenced by "a little friendly counsel and help," or even the infliction of "small fines." Among many other subjects dealt with in this report there is none more important than the inequality of sentences, on which some wise remarks are made. It is a crying evil, but one most difficult to remedy, because it must always, to a great extent, depend on the individual temperament of the judges and magistrates who have to decide the fate of criminals. The Lord Chief Justice has stated that there is a probability of a Royal Commission on the subject of sentences being appointed, and in August last the Home Secretary announced in Parliament that he was giving attention to the matter with a view to the appointment of such a committee. We are thankful, indeed, to know of this prospect, and it is much to be hoped that the measure will be put in force as soon as possible. The Howard Association has done good service in bringing the matter under public notice.

